# Treatment of a Maxillary First Molar with Two Palatal Roots

**DOI:** 10.7508/iej.2015.04.016

**Published:** 2015

**Authors:** Vahideh Asghari, Saeed Rahimi, Negin Ghasemi, Bita Talebzadeh, Ahmad Norlouoni

**Affiliations:** a *Department of Endodontics, Dental School, Tabriz University of Medical Sciences, Tabriz, Iran;*; b * Dental and Periodontal Research Center, Department of Endodontics, Dental School, Tabriz University of Medical Sciences, Tabriz, Iran*

**Keywords:** Anatomic Variation, Maxillary First Molar, Palatal Root, Root Canal Anatomy

## Abstract

Thorough knowledge of the morphology and internal anatomy of the root canal system is essential, because it determines the successful outcome of endodontic treatment. The main goal of endodontic treatment is to prevent apical periodontitis and/or to promote the healing of periapical lesion. Presence of two canals or roots on the palatal side of the first maxillary molar has rarely been reported. This case report presents a maxillary first molar with two separate palatal roots.

## Introduction

The main goal of endodontic treatment is to prevent apical periodontitis or in cases of existing lesion, to promote its healing [1, 2]. One of the main causes of endodontic treatment failure is the inability to negotiate, clean or obturate all the existing root canals [3]. Understanding the anatomical variations of the root canal system is essential in the success of endodontic treatment.

The permanent maxillary molars are generally described as a group of teeth with three roots including two buccal roots and one palatal root. The second mesiobuccal canal is a common finding [4, 5]. However, Shahi *et al.* [5] reported 0.73% of the first molars with two palatal canals and Zheng *et al.* [6] reported a prevalence rate of 1.12 and 1.17% for presence of an extra canal in the distobuccal and palatal roots, respectively.

Slowey [7] reported a maxillary second molar with two palatal roots for the first time. Unusual roots and root canal morphologies in the molars have been recorded in several studies [1, 2, 8, 9]. Christie *et al.* [10] reported 16 cases of maxillary molars and six extracted teeth with two palatal roots and classified them into three types as follows: *type* *I*; the buccal roots are often similar to cow horns and less divergent, the two palatal roots are very divergent and often long and tortuous, which can be observed radiographically, *type II*; the palatal roots are shorter and parallel and root apices are blunt, with mesial and distal divergence on the buccolingual radiographic view and *type III*; the roots have a constricted morphology with mesiobuccal, mesiopalatal and distopalatal roots engaged in a web-like radiographic view similar to *type II*. The distobuccal root remains isolated and may diverge in distobuccal direction.

It has been demonstrated that the patient's age plays an important role in the detection of fewer canals in maxillary molars, probably due to the calcification and morphological changes that occur with age, making it difficult to detect a maximum number of root canals [11]. There are some reports about two palatal roots in the second maxillary molars [12, 13].

Rostein and Libfeld [14] estimated a 0.4% incidence of two palatal canals for second maxillary molars in a large series of 1200 teeth using radiographic assessment. There are several case reports about the second maxillary molars with two palatal canals or roots [15-23]. Stone and Stroner [24] evaluated more than 500 extracted molars and found a prevalence rate of less than 2% for the presence of additional channels in the palatal roots of maxillary molars.

This case report presents a maxillary first molar with four roots, including two separate palatal ones, and one distobuccal and one mesiobuccal root.

**Figure 1:**
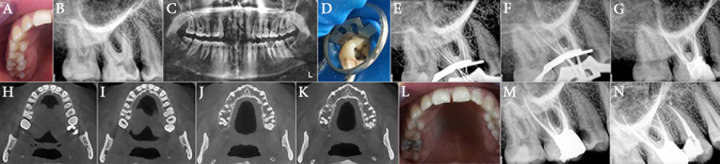
*A)* Preoperative photograph; *B)* Preoperative periapical radiograph; *C)* Panoramic view; *D)* Clinical photograph of the access cavity showing four root canals; *E)* Initial files; *F) *Master cones; *G)* Post-obturation periapical radiograph; *H to K)* CBCT images show four root canals in right maxillary first molar; *L)* Photographic view; *M)* Post-restoration radiograph of the tooth; *N)* Six-month follow-up radiograph

## Case Report

A 21-year-old woman was referred to the Department of Endodontics, Tabriz Faculty of Dentistry with a history of toothache which was elicited by exposure to cold water and during mastication in the right maxillary region. The patient’s medical history was non-contributory. Clinical examination revealed an extensive mesial carious lesion in the right maxillary first molar (Figure 1A) and tenderness to percussion, with positive response to vitality tests in the form of sever discomfort after cold testing. The tooth was diagnosed with symptomatic apical periodontitis and irreversible pulpitis. The preoperative radiographic views (periapical and panoramic) revealed two separate palatal roots, with normal periradicular tissue (Figures 1B and C).

The patient was prepared for endodontic treatment and received local anesthesia with 2% lidocaine containing 1:80000 epinephrine (Darupakhsh, Tehran, Iran). The conventional triangular access cavity was modified to a trapezoid shape to improve access to the extra canal and four orifices were found. Then a rubber dam was placed (Figure 1D). After removing the coronal pulp the working length of each canal was determined by means of Root ZX apex locator (J. Morita USA, Inc., Irvine, CA, USA) and confirmed by a radiograph (Figure 1E). The canals were navigated with #15 and #20 hand K-file (Dentsply Maillefer, Ballaigues, Switzerland) and then RaCe NiTi rotary files (FKG Dentaire, La-Chaux-de Fonds, Switzerland) were used with crown-down technique up to 30/0.04 as the master apical file buccal and palatal root canals. 

The root canals were irrigated with 2.5% NaOCl and normal saline during instrumentation. After a final rinse with normal saline the root canals were dried and the master cones (40/0.04) were adjusted (Figure 1F) and the canals were obturated with gutta-percha and AH-26 sealer (Dentsply, De Trey, Konstanz, Germany) using the lateral compaction technique (Figure 1G). Finally, the patient was referred to the restorative department for final restoration.

Cone-beam computed tomography (CBCT) of the upper jaw revealed no extra undiagnosed root canal (Figure 1H-K). After 6 months, the patient had no clinical symptoms and the tooth was functional and symptomless (Figure 1L-N).

## Discussion

The present manuscript reported a maxillary first molar with two separate palatal roots and two buccal roots that corresponded to Christie’s *type* *I* category [10].

The aim of root canal treatment is to achieve a clean root canal system and fill it in all the dimensions [2, 5]. Sometimes the root canals are not cleaned because the dentist fails to detect their presence. The variations in the anatomy of root canals have an important role in endodontic therapy [9, 25]. 

Anatomical variations happen frequently in maxillary molars. The occurrence of second maxillary molars with two palatal roots or two palatal root canals is reported to be 0.4-2% which is rather uncommon [9, 17]. It is interesting that the presence of double palatal roots/canals in the first maxillary molars is less common than the second maxillary molars [9]. The etiology of this condition is unclear. Formation of additional root(s) might be related to external factors intervening during odontogenesis or the emergence of atavistic genes [26]. Curzon [27] suggested that the presence of additional roots in molars is most likely related to genetic penetrance. 

Evaluation of the radiographs is necessary to detect the anatomical variations of maxillary molars. Careful evaluation of unusually massive coronal morphology is necessary during clinical examination [17].

To detect additional roots, different methods should be used besides normal procedural protocols; the dentist should look for the following signs which might show the presence of additional roots or canals [20]:

***1.*** ***Evaluation of*** ***multiple radiographs:*** Radiographs should be taken at different angles.

***2. Digital radiography:*** This is recommended due to the diversity of software, especially in the diagnosis of un-detected calcified or un-treated canals.

***3. Coronal flaring:*** It would be better to visualize the canal orifices.

***4. White line test:*** The pulpal floor meets the dentinal walls and creates a groove which can be followed to detect canal orifices.

***5. Red line test:*** In vital teeth the remaining blood in the orifices, fins and isthmi can help identifying the canal orifices.

***6. Piezoelectric ultrasonic device:*** The grooves should be followed with ultrasonic tips.

***7. Troughing:*** The floor of the pulp chamber should be examined with a sharp explorer.

***8. Champagne bubbling test*** ***with*** ***sodium*** ***hypochlorite:*** After cleaning and shaping, the access cavity should be filled with NaOCl solution and observed to see if bubbles form at the pulp chamber floor; this phenomenon is indicative of proteolytic activity of the NaOCl solution and shows the presence of remaining tissue within the undiscovered canals. 

***9. Dyes: ***Also a non-toxic dye such as 1% methylene blue, can be brushed over the chamber floor and then washed with water. Commonly it will be absorbed into orifices, fins and isthmus areas [20].

***10. Armed eyes:*** Loupes or dental operating microscopes (DOM) can be very helpful [28-30] .

***11. Additional imaging techniques:*** CBCT images always help finding greater number of roots or canals compared to traditional methods. In our case CBCT was used.

Teeth with two palatal roots often have wider mesiodistal dimension; therefore, the access cavity should be wider on the lingual side than usual and the access outline must be square instead of triangular [3]. The prognosis of treatment in these teeth should be similar to any other endodontically treated teeth [8].

## Conclusion

Anatomic variations in root canal morphology especially in molars, is a challenge for endodontic therapy. Endodontists should be aware of these variations making sure that they have not missed the second canal/root on the palatal side.
